# A meta-analysis suggests the association of reduced serum level of vitamin D and T-allele of Fok1 (rs2228570) polymorphism in the vitamin D receptor gene with celiac disease

**DOI:** 10.3389/fnut.2022.996450

**Published:** 2023-01-19

**Authors:** Tanya Shree, Pratibha Banerjee, Sabyasachi Senapati

**Affiliations:** Immunogenomics Laboratory, Department of Human Genetics and Molecular Medicine, School of Health Sciences, Central University of Punjab, Bathinda, Punjab, India

**Keywords:** celiac disease, vitamin D deficiency, vitamin D receptor, Fok1 polymorphism, meta-analysis, autoimmunity

## Abstract

**Purpose:**

As an immune-modulator, vitamin D is known to regulate immune response and is implicated in disease pathogenesis. Celiac disease (CD) is a systemic autoimmune disease and susceptibility conferred by vitamin D metabolism is under investigation. Studies on the association of vitamin D metabolism and genetic polymorphisms are expected to explain CD pathogenesis. We performed a systematic review–based meta-analysis to investigate the 25(OH)D serum levels and susceptibility conferred by the genetic variants of VDR in CD.

**Methods:**

Systematic review was conducted through a web-based literature search following stringent study inclusion–exclusion criteria. The Newcastle–Ottawa Scale and GRADE tools were used to assess the quality of evidence in studies and the study outcome. Cohen's κ value was estimated to access the reviewer's agreement. RevMan 5.4.1 was used to perform the meta-analyses. Weighted mean difference and Meta *p*-value was assessed for 25(OH)D serum levels. Meta-odds ratio and *Z*-test *p*-value were evaluated to estimate the allelic susceptibility of *VDR* variants.

**Results:**

A total of 8 out of 12 studies were evaluated for “25(OH)D” serum level, while four studies were found eligible for SNPs (*Bsm1, Apa1, Fok1*, and *Taq1*) of *VDR*. Significantly higher levels [WMD = 5.49, *p* < 0.00001] of 25(OH)D were observed in healthy controls than in patients with CD. rs2228570-T (*Fok1*) [Meta-OR = 1.52, *p* = 0.02] was confirmed to be predisposing allele for CD.

**Conclusion:**

Reduced serum level of 25(OH)D and association of *Fok1* T-allele of *VDR* confirmed in this study plays a critical role in immunomodulation and maintaining barrier integrity, which is majorly implicated in CD.

## 1. Introduction

Celiac disease (CD) is an immune-mediated gluten enteropathy affecting almost 1% of the population worldwide in individuals carrying HLA DQ2/8 susceptibility alleles, which are encoded by DQA1^*^0501-DQB1^*^02 and DQA1^*^0301-DQB1^*^0302 (1, 2). Almost 94.94% of the CD subjects are positive for this specific HLA-DQB1^*^02 allele (2). Though the etiology of CD is not well understood but is marked by the presence of inflammatory cytokines IL18, IL17, TNF-α, IL12, IL21, and IL15 (3). Vitamin D was found to be associated with reducing the effects of inflammatory molecules (4). Components of the immune system, such as B-lymphocytes, T-lymphocytes, and dendritic cells, are influenced by the regulatory effects of vitamin D and expressed vitamin D receptor (VDR), which is involved in the biological activity of 1,25(OH)_2_D3, and these cells also have the capability of locally synthesizing active 1,25(OH)_2_D3 (5). This active form of vitamin D exerts its effects by binding to the nuclear receptor VDR. The 1,25(OH)_2_D3-VDR complex dimerizes with the retinoid X receptor (*RXR*), and the 1,25(OH)_2_D3-VDR-RXR heterodimer translocates to the nucleus where it binds Vitamin D Response Element (VDRE) in the promoter regions of vitamin D responsive genes and induces the expression of vitamin D responsive genes (6). Some of the remarkable effects of vitamin D in immune system regulation were the suppression of Th1/Th17 CD4+ T cell proliferation and subsequent alteration of the cytokine responses (5). Thus, it is worth studying the association between vitamin D metabolism and various immune-mediated disorders.

Duodenal epithelial damage in CD is caused by the cytokines released by the activated T-cells upon exposure to gliadin peptides, and vitamin D is reported to suppress the proliferation of T-cells (7, 8). *In vitro* study performed on Caco-2 cell layers reported the protective role of 1,25(OH)_2_D3 on the damage of tight junction, which was induced by the pepsin–trypsin digested gliadin (PT–G). Vitamin D was observed to increase the expression of the tight junction–associated proteins and was also able to minimize epithelial permeability (9). Notably, vitamin D deficiency increases the risk of severe intestinal damage, which is a prominent symptom of CD (10). During the early infant stage, 25(OH)D concentrations between <30 and >75 nmol/L were associated with an increased risk of developing CD in genetically predisposed children. The non-linear relationship raises the need for more studies on the possible role of 25(OH)D in the onset of CD (11). *In vivo* studies have shown a positive response to vitamin D supplementation in the celiac mice model (12).

In the association study in the Spanish Basque population, four polymorphisms of *VDR* (*Bsm1*-rs1544410, *Apa1*-rs7975232, *Taq1*-rs731236, and *Fok1*-rs2228570) were genotyped. *Fok1* was reported to be a risk genotype in 25.64% of CD cases as compared to 9.89% of controls (*p* = 0.01, OR = 3.45) (13). Another association study on the Norwegian cohort did not find any association with the *VDR* marker (*Bsm1*) or serum vitamin D level (14). Two significant SNPs, *Fok1* and *Bsm1*, were reported in the Russian Tomsk group, *p* = 0.009 and *p* = 0.001, respectively (15). A total of 92 Viennese CD patients and controls were genotyped for *Apa1* and *Taq1* SNPs of the *VDR* gene; however, no significant risk association was observed (16). A recent meta-analysis study by Lu et al. (17) did not find any association with VDR genotype but reported lower levels of 25(OH)D in CD patients than in controls.

In this systematic review and meta-analysis, we intended to assess an association of serum level of 25(OH)D and *VDR* gene polymorphism with CD. Four SNPs (*Bsm1*-rs1544410, G>A; *Apa1*-rs7975232, C>A; *Taq1*-rs731236, T>C; and *Fok1*-rs2228570, C>T) of *VDR* were evaluated.

## 2. Methods

### 2.1. Search strategy

For the search and retrieval of relevant published literature, various web search engines for scientific databases such as Google scholar, NCBI (PubMed/MEDLINE), SCOPUS, EMBASE, and Web of Science were used. All the published literature until May 2022 on the association of vitamin D serum concentration and *VDR* gene polymorphism with celiac disease was searched. For this literature search, keywords were used as follows: “Celiac disease” AND “serum vitamin D concentration” OR “serum 25(OH)D concentration”. For the genotype association study, the key terms used were as follows: “Celiac disease” AND “*VDR* polymorphism” OR “*VDR* genotype” OR “*VDR* variants” OR “*Bsm1* polymorphism” OR “*Apa1* polymorphism” OR “*Fok1* polymorphism” OR “*Taq1* polymorphism”.

### 2.2. Inclusion and exclusion criteria

To limit the screening to relevant articles, the inclusion and exclusion criteria were defined. Articles were considered eligible for the study if they met the following inclusion criteria: (i) published full-text original research article, (ii) case–control study design, (iii) mean serum 25(OH)D concentration can be obtained, (iv) *VDR* gene polymorphism association study with celiac disease and healthy controls, and (v) Only articles written in the English language. Our study was not restricted to any specific population or ethnicity, and all the relevant articles available till May 2022 were considered.

The exclusion criteria for the study were as follows: (i) studies other than case–control, (ii) if the CD patients were on a gluten-free diet or vitamin D supplementation, and (iii) review articles. Apart from this, duplicate publications were also excluded.

### 2.3. Data extraction and evaluation of confined studies

Two researchers independently performed a literature search checked the eligibility criteria, and extracted data from the shortlisted literature. From all the studies which we considered eligible according to our inclusion and exclusion criteria, relevant data for serum vitamin D concentration and *VDR* genotype in patients with CD and healthy controls were retrieved along with the name of the author and the year of publication. For the concentration of vitamin D, the serum levels of 25(OH)D [mean ± standard deviation (SD)] in patients with CD and healthy controls were extracted from every eligible article, and for the vitamin D concentration, the units considered were in ng/ml, data obtained in other units (such as nmol/L) were converted to ng/ml (1 ng/ml = 2.5 nmol/L). Few other information about the study population such as the mean age of the patients with CD and healthy controls and the male:female ratio in the study were also obtained.

For the *VDR* genotype association studies, *VDR* SNPs data of CD cases and controls were obtained. Four SNPs of the *VDR* gene: *Bsm1*-rs1544410, *Apa1*-rs7975232, *Fok1*-rs2228570, and *Taq1*-rs731236 were analyzed, and the genotype frequencies in CD cases and controls were obtained. After data extraction, to carry out a systematic review, standard checklists were used to analyze methodological quality and strength of association, which also included the risk of bias evaluation in observational studies as recommended by the Cochrane handbook (https://training.cochrane.org/handbook). The Preferred Reporting Items for Systematic Reviews and Meta-analysis (PRISMA) statement was followed for reporting the findings (http://www.prisma-statement.org/) (18). The PRISMA statement is provided in Supplementary Table 1.

### 2.4. Quality assessment

Cohen's kappa (κ) value was calculated in order to estimate the extent of concordance between two reviewers, who performed a literature search, and checked eligibility and data extraction (19). Based on the percentage of agreement and Cohen's κ score, values were classified as poor, slight, fair, moderate, substantial, or almost perfect. Sensitivity analysis was used to evaluate the impact of each study on the meta-analysis result by removing one study at a time from the combined dataset. Funnel plots were analyzed in order to detect any study biasness. If any of the studies fell outside the funnel plot or gave rise to an asymmetric funnel plot, then that study was considered to be biased. Irrelevant studies were ruled out after a thorough analysis. The Newcastle–Ottawa Scale (NOS) tool was used to assess the quality of the eligible studies (i.e., study participant selection, comparability, and outcome) (20). Studies were graded on a scale of 0 (lowest) to 9 (highest), with low (stars 7–9), moderate (stars 4–6), and high (stars 0–3) risk of bias. The analysis was limited to studies with low risk.

GRADEpro.v.3.6 was used to assess the quality of evidence (QoE) for each of the outcomes using The Grades of Research, Assessment, Development, and Evaluation (GRADE) tool (21). Based on the proposed criteria, the included study design, risk of bias, inconsistency, indirectness, imprecision, and publication bias, and evidence were grouped into four main categories: high, moderate, low, and very low.

### 2.5. Statistical analysis

For the meta-analysis of the serum 25(OH)D level, the mean difference was used to analyze the pooled continuous data of serum 25(OH)D concentration which was obtained as mean ± standard deviation (SD). To evaluate the combined MD, mean differences (MD) with 95% confidence intervals (CIs) were presented for all relevant studies on a forest plot. A collective study was carried out without dividing the study based on age group due to very few studies were obtained with relevant data in the adult age group. The dichotomous data of the alleles of the four SNPs of the *VDR* gene were utilized to calculate Meta-OR using Mantel–Haenszel (M–H) method with 95% CI). The statistical analysis for this study was carried out using the RevMan (version 5.4.1, The Cochrane Collaboration) software. For this meta-analysis, the assessment of study heterogeneity was done by chi-square *p*-value and *I*^2^ value. The meta-analysis was carried out using the DerSimonian and Laird random effect model for *I*^2^ > 50% and *p* < 0.05, and for *I*^2^ < 50% and *p* > 0.05, the fixed effect model was used.

## 3. Results

### 3.1. Features of enclosed studies

**Figure 1:**
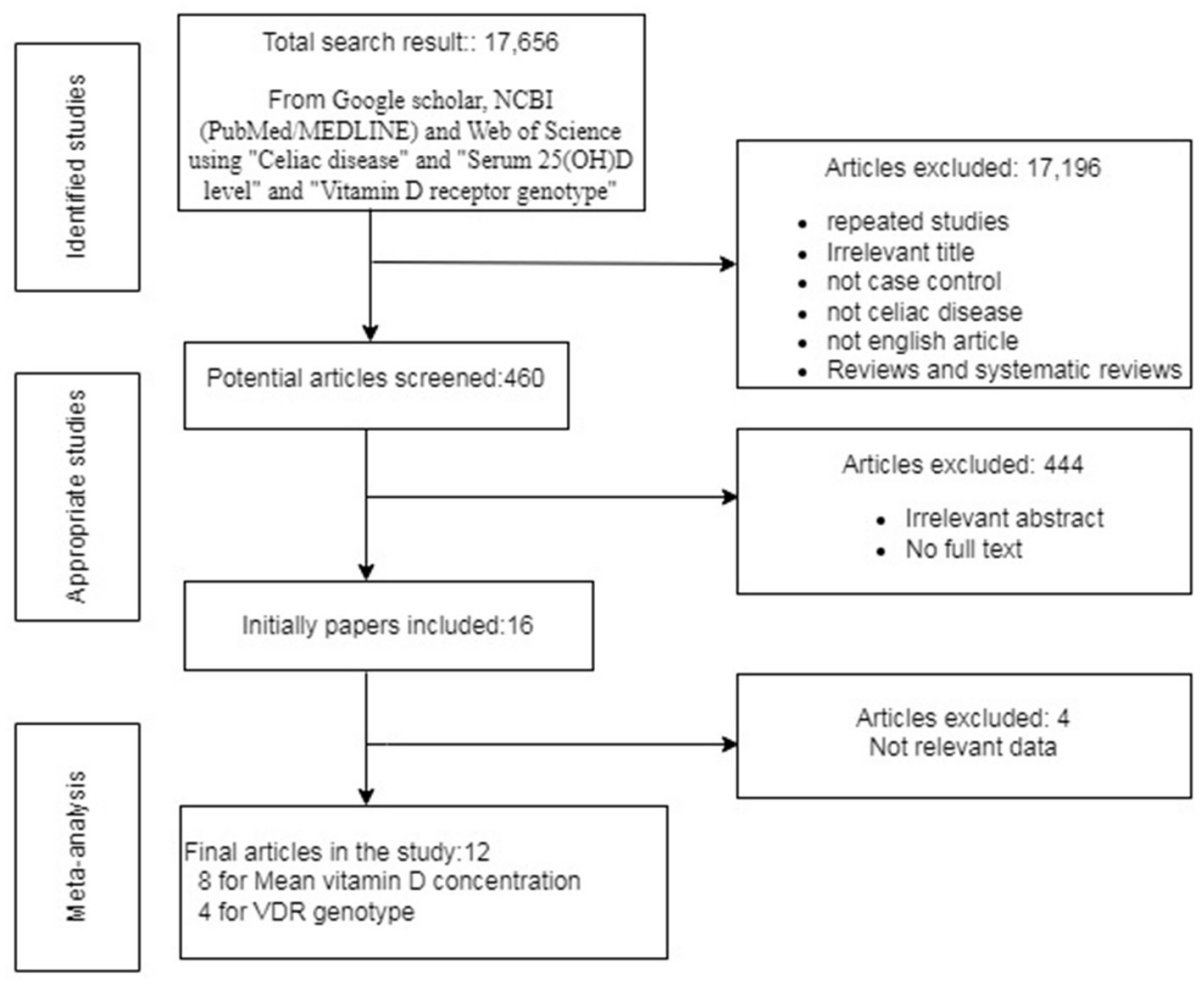
Flow chart exhibiting the inclusion of eligible studies for meta-analysis.

After employing all the above-described literature retrieval strategies, we got a total of 17,656 studies. Overall, 17,196 studies were excluded based on the inclusion and exclusion criteria because of irrelevant titles or because they were review articles or were not in the English language. After screening the rest of the articles, 444 articles were excluded from our study because of duplicate publications, irrelevant abstracts (*in silico, in vitro, in vivo* studies, case studies), and articles without full text. Initially, a total of 16 studies were considered for this study but due to the lack of relevant data, four articles were excluded. Finally, 12 studies that satisfied our inclusion and exclusion criteria and had the data of serum 25(OH)D level and *VDR* genotype in CD cases and control were included in this meta-analysis as shown in Figure 1. All these data of mean serum 25(OH)D concentration and the genotype of *VDR* SNPs *Bsm1* rs1544410, *Apa1* rs7975232, *Fok1* rs2228570, *Taq1* rs731236, and their summary statistics are provided in Tables 1, 2.

**Table 1:** Summary table of the alleles included in the meta-analysis and the quality of evidences as graded by the GRADE tool.

**Gene**	**Marker (Allele)**	**Overall study comparison**					**Assessment of quality of evidence(GRADE Tool)**							
		**N**	**Total CD cases**	**Total Healthy Controls**	**Ref Allele Meta-OR (95% CI)**	**I**^2^ **(%) p-value**	**Study design**	**Risk of bias**	**Inconsistency**	**Indirectness**	**Imprecision**	**Publication bias**	**Quality of evidence**	**Importance**
VDR	Bsm1 rs1544410	3	964	1468	0.98[0.83,1.16]	37% (p = 0.84)	Non-randomized observational case-control	Not serious	Not serious	Not serious	Not serious	None	Low	Important
	Apa1 rs7975232	2	262	398	1.21[0.61,2.38]	76% (p = 0.58)	Non-randomized observational case-control	Not serious	Not serious	Not serious	Not serious	None	Low	Important
	Fok1 rs2228570	2	176	402	1.52[1.06,2.18]	0% (p = 0.02)	Non-randomized observational case-control	Not serious	Not serious	Not serious	Not serious	None	Low	Important
	Taq1 rs731236	2	262	404	0.78[0.46,1.32]	57% (p = 0.35)	Non-randomized observational case-control	Not serious	Not serious	Not serious	Not serious	None	Low	Important

#, Quality of evidence was accessed using six parameters. QoE was downgraded (by 1 or 2 depending on the severity) for the following: study design- randomized controlled trials are preferred over non-randomized/observational/case-control studies; risk of bias-downgraded for weak study design, short follow-up, and not matched case controls; inconsistency-downgraded for considerable heterogeneity, direction of effect, and lack of replication; indirectness-downgraded when population and diagnostic criteria varies; imprecision-wide confidence of interval and optimal information size; publication bias-observation from funnel plots.

High quality, Further research unlikely change the study findings and effect estimates; Moderate quality, Further research is likely to change the study findings or the effect estimates; Low quality, Further research is very likely to have an impact on the confidence and effect estimates; Very low quality, Uncertain estimates; #, Primarily influenced by the study design alone; *, Beside study design, one or two factors are implicated.

**Table 2:** Summary table of mean 25(OH)D concentration included in the meta-analysis and the quality of evidences as graded by the GRADE.

	**Overall study comparision**					**Assessment of quality of evidence (GRADE Tool)**							
**Serum concentration**	**N**	**Total CD cases**	**Total healthy controls**	**Mean-difference (95% CI)**	**I**^2^ **(%) p-value**	**Study design**	**Risk of bias**	**Inconsistency**	**Indirectness**	**Imprecision**	**Publication bias**	**Quality of evidence**	**Importance**
25(OH)D	8	592	754	5.49 [3.22, 7.76]	73% (*p* = 0.0001)	Non-randomized Observational Case-Control	Not serious	Not serious	Not serious	Not serious	None	Low	Important

#, Quality of evidence was accessed using six parameters. QoE was downgraded (by 1 or 2 depending on the severity) for the following: study design- randomized controlled trials are preferred over non-randomized/observational/case-control studies; risk of bias-downgraded for weak study design, short follow-up, and not matched case controls; inconsistency-downgraded for considerable heterogeneity, direction of effect, and lack of replication; indirectness-downgraded when population and diagnostic criteria varies; imprecision-wide confidence of interval and optimal information size; publication bias-observation from funnel plots. High quality, Further research unlikely change the study findings and effect estimates; Moderate quality, Further research is likely to change the study findings or the effect estimates; Low quality, Further research is very likely to have an impact on the confidence and effect estimates Very low quality, Uncertain estimates; #, Primarily influenced by the study design alone; *, Beside study design, one or two factors are implicated.

### 3.2. Publication bias evaluation and sensitivity analysis

In order to detect any study biasness in this meta-analysis, the funnel plots of all the studies were analyzed. Upon this analysis, the exclusion of any study was not done as study biasness was not detected. When it came to the inclusion and exclusion of relevant and irrelevant articles from this systematic review, reviewers were almost unanimous (Cohen's κ = 0.96; % agreement 98.28). All the 12 eligible studies included in this meta-analysis are given in Supplementary Figure 1.

### 3.3. Quality of the studies included and risk of biasness

Based on the evaluation of the quality of evidence, all 12 studies were identified to have a low risk of bias (NOS = 7–8) (Supplementary Table 2). Because of concise criteria for evaluation and homogeneous populations, GRADE's approach observation indicated that none of the 12 studies increased the possibility of bias, and the indirectness of the findings was not a concern. The evidence quality was insufficient to rule out any of the studies that were included. As a result, the entire study was deemed important and rated as low risk in Supplementary Table 2.

### 3.4. Concentration of 25(OH)D in CD patients and control

**Figure 2:**
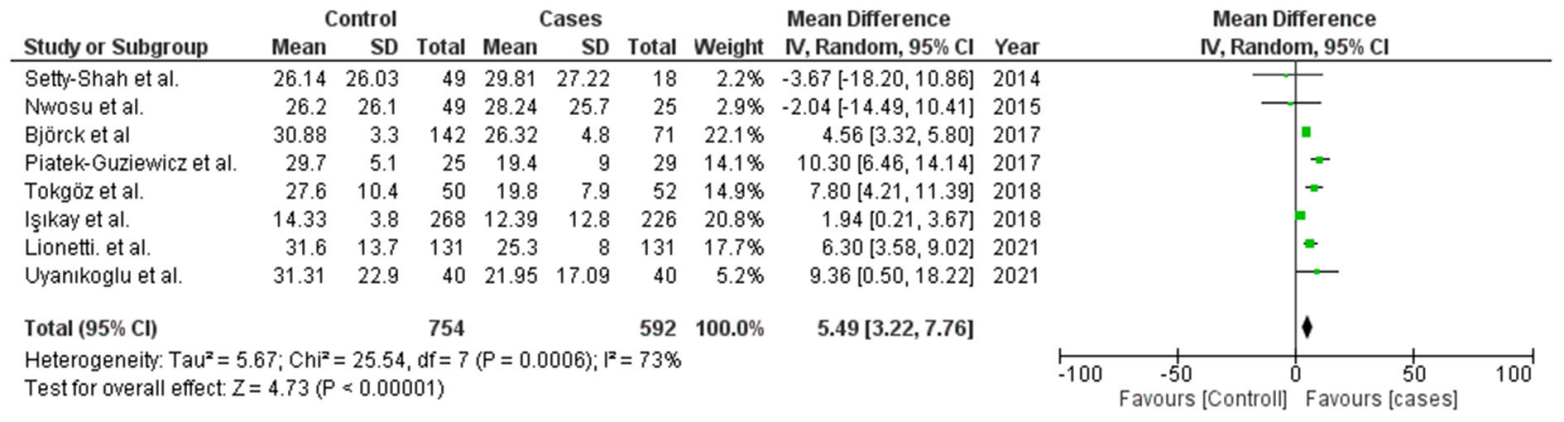
Forest plot to show meta-analysis of vitamin D serum concentration in CD patients and control.

A total of eight studies were evaluated in this meta-analysis with mean serum 25(OH)D concentration of patients with CD and controls, the list of same is given in Supplementary Table 3 (22–29). It constitutes 592 patients with CD and 754 controls. This meta-analysis was carried out using the Random effect model. Significant associations were defined as Z-test *p*-values (i.e., Meta-*p*-values) of <0.05. The mean difference was evaluated for all the studies included, and a forest plot was plotted using the mean differences with 95% CI, of each of these studies to estimate the combined mean difference (Figure 2). The outcome of this meta-analysis exhibited that the mean 25(OH)D concentration in the healthy controls was 5.49 ng/ml higher than that of CD patients (WMD = 5.49, 95% CI = 3.22–7.76). The observed meta-*p*-value was significant (*p* < 0.00001) (Figure 2).

### 3.5. VDR allele association with CD patients and controls

For this meta-analysis of allelic association, four studies were included in which the allelic frequencies for the four *VDR* SNPs (*Bsm1* rs1544410, *Apa1* rs7975232, *Fok1* rs2228570, and *Taq1* rs731236) were given for patients with CD and healthy controls, basic information of which is provided in Supplementary Table 4. The meta-analysis for the SNPs *Bsm1* rs1544410 and *Fok1* rs2228570 was performed using the fixed effect model for insignificant heterogeneity (*I*^2^ < 50% and *p* > 0.05), whereas for *Apa1* rs7975232 and *Taq1* rs731236, the random effect model was used because of a high degree of data heterogeneity. Significant associations were defined as Z-test *p*-values (i.e., Meta-*p*-values) <0.05. The Meta-OR was used to predict risk.

#### 3.5.1. Bsm1

**Figure 3:**
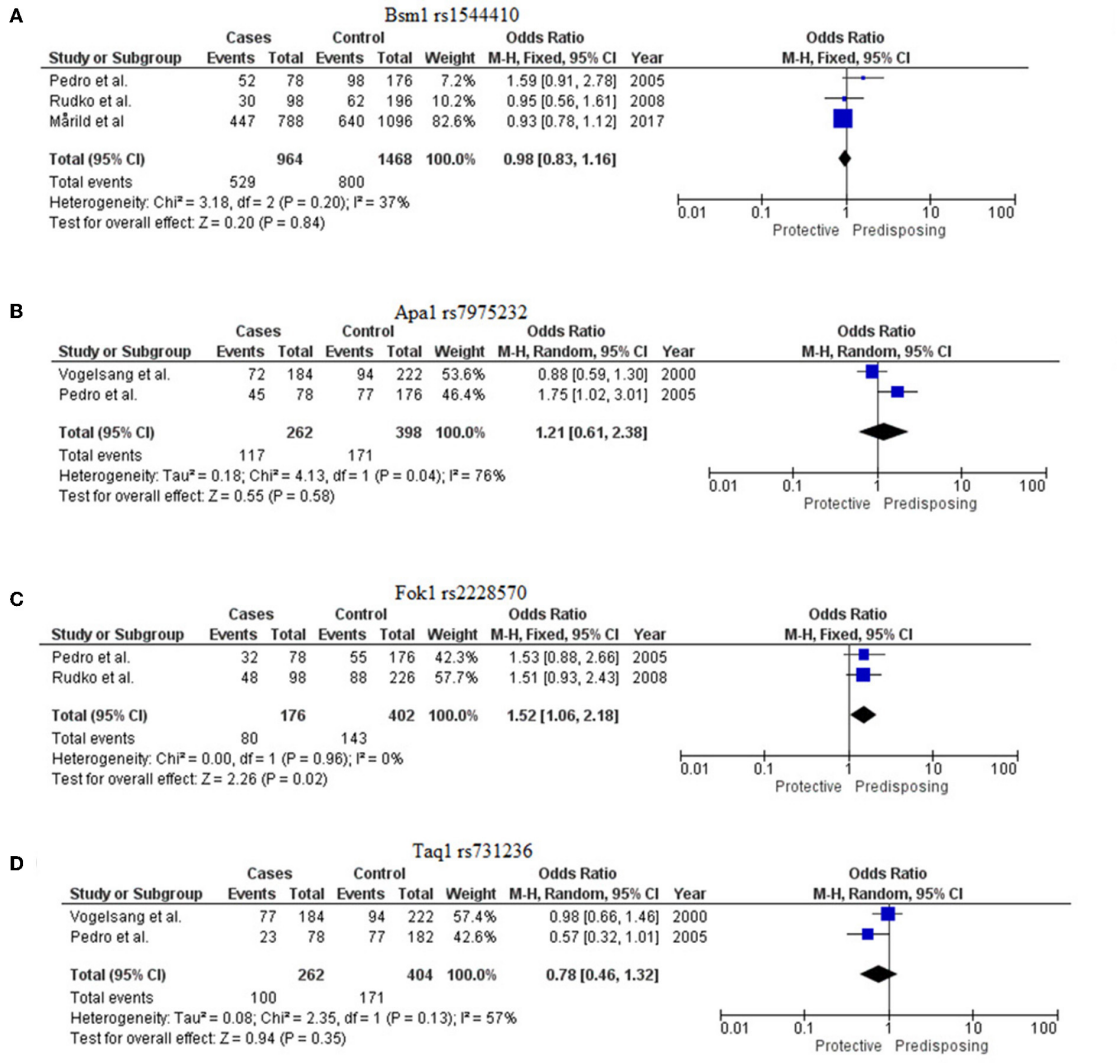
Forest plots to show meta-analysis of VDR SNPs [**(A)** *Bsm1 rs*1544410, **(B)** *Apa1 rs*7975232, **(C)** *Fok1 rs*2228570, and **(D)** *Taq1 rs*731236].

Three studies were evaluated (13–15). The G allele of this marker rs1544410 was found to be protective for CD [Meta-OR = 0.98 (0.83–1.16), *p* = 0.20]. The meta *p*-value was declared insignificant (Figure 3A).

#### 3.5.2. Apa1

Two studies were considered for this *VDR* marker (13, 16). The C allele of this marker rs7975232 was found to confer risk for CD [Meta-OR = 1.21 (0.61–2.38), *p* = 0.58] but with insignificant *p*-value (Figure 3B).

#### 3.5.3. Fok1

Two eligible studies were included for this marker (13, 14). The T allele of rs2228570 was identified to be significantly predisposing for the disease [Meta-OR = 1.52 (1.06–2.18), *p* = 0.02] (Figure 3C).

#### 3.5.4. Taq1

Two studies were evaluated for this meta-analysis (13, 16). T allele of rs731236 was found to be protective [Meta-OR = 0.78 (0.46–1.32), *p* = 0.13]. But no significant association was found (Figure 3D).

## 4. Discussion

Multisystem CD is characterized by circulating innate lymphoid cells and increased levels of IL-18, IFN-γ, and innate lymphoid cell precursors were noted (3). A reduced vitamin D level has been reported to be correlated with higher IFN-γ and innate lymphoid cell precursor (4). Vitamin D deficiency is shown to induce T-cell-mediated pro-inflammatory immune responses that are pivotal in CD (30, 31). This gave an insight into dietary supplementation of vitamin D as a therapeutic approach to inhibit cytokine IFN-γ production (4). Several cross-section studies concluded the association of vitamin D deficiency with immune-related diseases (32–34). *In vitro* and *in vivo* studies on induced CD-like conditions reported vitamin D supplementation rescue from cellular and tissue damage, which directly indicated the protective role of vitamin D in CD (8, 35, 36).

**Figure 4:**
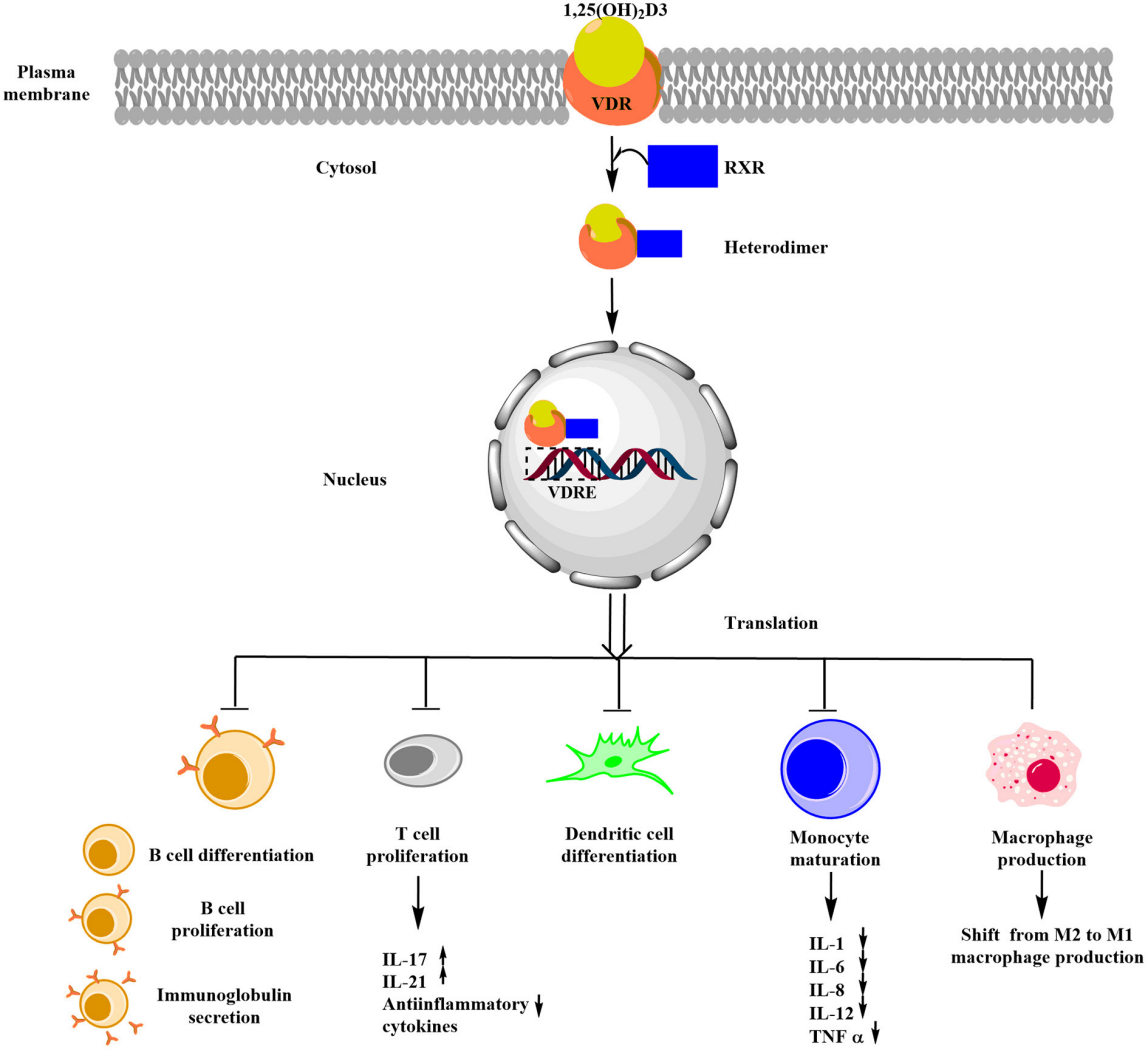
Schematic representation of the role of vitamin D in immune regulation in CD. Upon binding of vitamin D3 with VDR, RXR is recruited and results in the formation of a heterodimer known as VDR-RXR complex, whose translated product (formed by binding of the heterodimer with VDRE) exerts immune modulation by inhibiting differentiation and proliferation of B-cell, T-cell, and dendritic cells and also inhibits immunoglobulin secretion.

This meta-analysis suggests the association of the reduced serum level of 25(OH)D [MD = 5.49; *P* < 0.00001] and rs2228570-T (*Fok1*) [Meta-OR = 1.52, *p* = 0.02] with CD. All the studies were performed in the last decade and on a modest sample size (Figure 2). Cross-section studies with case-control study design, which was included in this meta-analysis, are however unable to comment on the cause-effect relationship between VDD and CD. Nevertheless, this finding suggests vitamin D supplements to subjects with CD to restore the normal duodenal mucosal barrier and suppress inflammatory immune responses as illustrated in Figure 4. 25(OH)D is the most stable form of vitamin D, and its transport and stability are determined by the availability of vitamin D binding protein (VDBP) in the serum. To date, no reports are available on the association of serum VDBP with CD.

The extra-calcium role of vitamin D and its involvement in immune modulation suggested that in genetically predisposed individuals, vitamin D deficiency can be an underlying cause for the onset of CD in children. Moreover, vitamin D deficiency can lead to dysregulated immune responses that result in abnormal intestinal mucosa and a greater risk of developing acute gastrointestinal infection (37). Several reports are available to suggest significant improvements in subjects with CD following vitamin D supplementation alongside gluten-free diet (GFD) (38).

Very limited genetic association studies were performed on CD to determine the contribution of vitamin D metabolism to the disease. Only four research articles were available on VDR, and all the studies were performed in populations with European ancestry (Supplementary Table 4). Four polymorphisms namely, *Bsm1* (rs154441, G>A), *Apa1* (rs7975232, C>A), *Fok1* (rs2228570, C>T) and *Taq1* (rs731236, T>C) were considered where at two studies were available for the meta-analysis. The association of the *Fok1*-T allele with CD (OR = 1.52, *p* = 0.02) suggested the putative role of this gene in the disease pathogenesis (Figure 3C).

The *Fok1* polymorphism of *VDR* is associated with several other immune-mediated disorders such as type 2 diabetes (T2DM) (39). The *Fok1* polymorphism also known as the start codon polymorphism (SCP), in exon 2 of the *VDR* has been shown to alter the structure of the VDR. The change in C > T also represented as F > f leads to a threonine to methionine substitution and provides two possible sites for the initiation of translation (40). The shorter VDR form, that is, 424 amino acid protein (encoded by the common allele C) in the FF genotype appears to be more effective in binding 1,25(OH)_2_D_3_ and has a higher binding capacity (41), while rs2228570-T (in ff genotype) leads to the production of 427 amino acid protein product, which is comparatively 1.7 times less efficient at the binding of 1,25(OH)_2_D_3_. Reduced binding efficiency with 1,25(OH)_2_D_3_ thus restricts VDR activation, and therefore, may limit regulating the expression of specific genes that are implicated in immune regulation.

## 5. Conclusion

In this meta-analysis, lower levels of serum 25(OH)D were observed in patients with CD, which indicates that deficiency of vitamin D may play a significant role in the pathogenesis of CD. The SNPs of the *VDR* gene (*Bsm1*-rs1544410, *Apa1*-rs7975232, and *Taq1*-rs731236) did not show any significant association with CD, but *Fok1* (rs2228570-T) was identified to be providing significant risk for CD. However, due to limitations in the number of studies performed on the association of *VDR* gene polymorphism and CD, strong evidence to support this association is still lacking.

## Data availability statement

The original contributions presented in the study are included in the article/Supplementary material, further inquiries can be directed to the corresponding author.

## Author contributions

SS conceptualized the study. SS and TS designed the study and performed the initial literature search. PB and TS performed the systematic review. SS and PB performed the analysis and wrote the manuscript. All the authors read the final manuscript and approved it for publication.

## Supplementary material

Supplementary Figure 1 Funnel plot analysis of all eligible studies for serum vitamin D concentration and VDR gene SNPs.

Supplementary Table 1 PRISMA checklist.

Supplementary Table 2 The Newcastle-Ottawa Scale (NOS) assessment for all the twelve studies found eligible for this study ensuring the quality of evidence.

Supplementary Table 3 Mean difference of vitamin D [25(OH)D] concentration in serum.

Supplementary Table 4 Allele frequencies of four VDR SNPs those were included in the study of healthy controls and CD patients.
