# Supplementary figures and images for: A meta-analysis suggests the association of reduced serum level of vitamin D and T-allele of Fok1 (rs2228570) polymorphism in the vitamin D receptor gene with celiac disease
Source: Front Nutr. 2023 Jan 19;9:996450. doi: 10.3389/fnut.2022.996450 (PMC9893277; doi:10.3389/fnut.2022.996450)

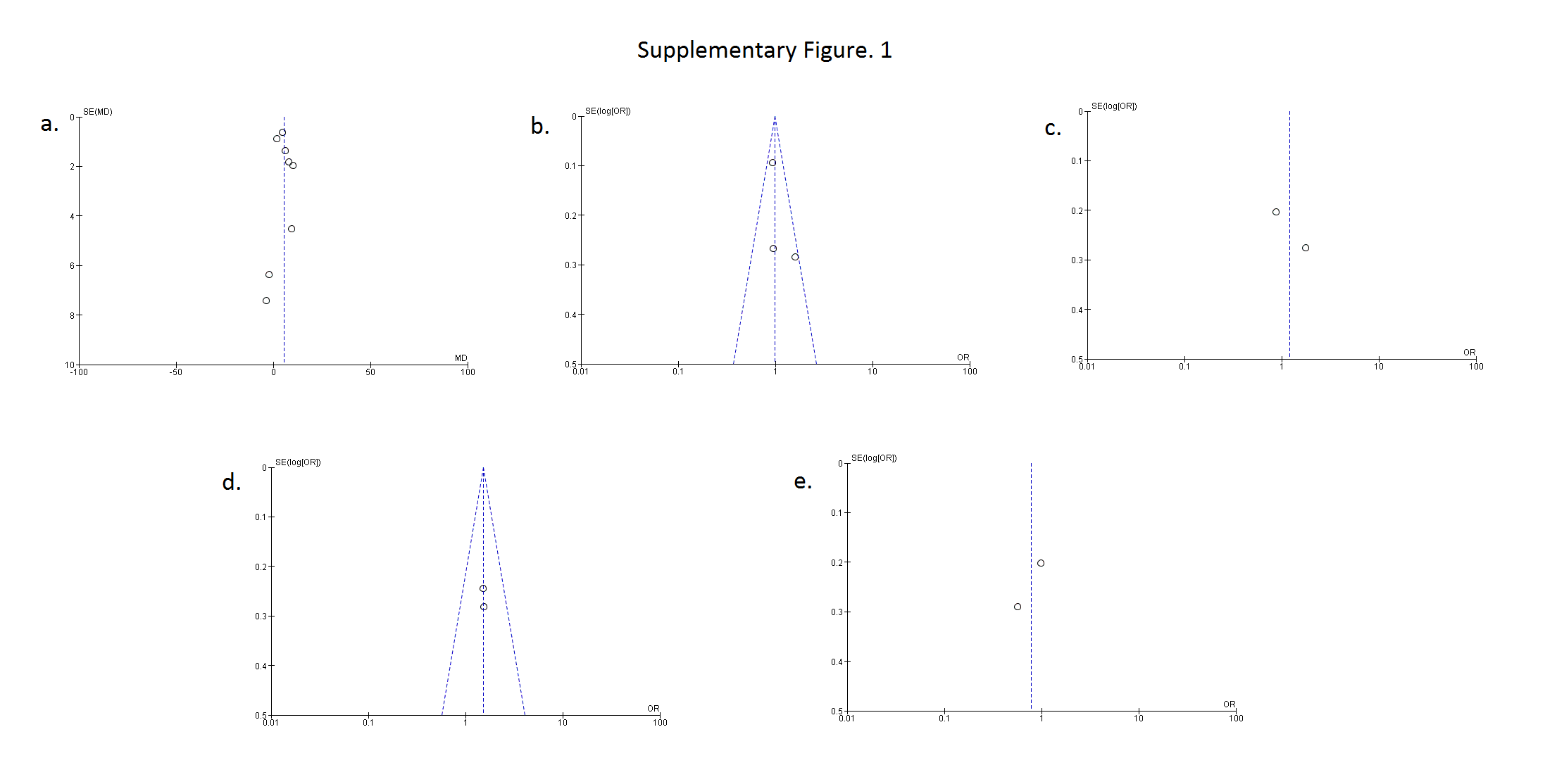

Supplement: Supplementary Figure 1 — Funnel plot analysis of all eligible studies for serum vitamin D concentration and VDR gene SNPs. [file Image_1.TIFF]
