# Supplementary material for: A meta-analysis suggests the association of reduced serum level of vitamin D and T-allele of Fok1 (rs2228570) polymorphism in the vitamin D receptor gene with celiac disease
Source: Front Nutr. 2023 Jan 19;9:996450. doi: 10.3389/fnut.2022.996450 (PMC9893277; doi:10.3389/fnut.2022.996450)
Supplement: Supplementary Table 2 — The Newcastle-Ottawa Scale (NOS) assessment for all the twelve studies found eligible for this study ensuring the quality of evidence. [file Table_2.docx]

**Supplementary Tables**

**Supplementary Table 2:** The Newcastle-Ottawa Scale (NOS) assessment for all the twelve studies found eligible for this study ensuring the quality of evidence

| **Study** | **PMID** | **Selection** | **Comparability** | **Exposure** | **Total NOS Star rating** | **NOS assessment** |
| --- | --- | --- | --- | --- | --- | --- |
| Setty-Shah et al. 2014 | 25548555 | 1) Is the case definition adequate? (a) Yes, with independent validation *  2) Reperesentativeness of the cases (a) Consecutive or obviously representative series of cases*  3) Selection of controls (a) Community control*  4) Definition of controls (a) No history of disease (endpoint)* | 1.) Comparability of cases and controls on the basis of the design or analysis:  (a) Study controls for serum 25(OH)D concentration CD.* | 1) Ascertainment of exposure: (a) Secure record *  2) Same method of ascertainment for cases and controls * (b) Yes  3) Non-Response rate (a) Same rate for both groups* | 8 | very low risk |
| Nwosu et al. 2015 | 26043850 | 1) Is the case definition adequate? (a) Yes, with independent validation *  2) Reperesentativeness of the cases (a) Consecutive or obviously representative series of cases*  3) Selection of controls (a) Community control*  4) Definition of controls (a) No history of disease (endpoint)* | 1.) Comparability of cases and controls on the basis of the design or analysis:  (a) Study controls for serum 25(OH)D concentration CD.* | 1) Ascertainment of exposure: (a) Secure record *  2) Same method of ascertainment for cases and controls * (b) Yes  3) Non-Response rate (a) Same rate for both groups* | 8 | very low risk |
| Björck et al. 2017 | 28319607 | 1) Is the case definition adequate? (a) Yes, with independent validation *  2) Reperesentativeness of the cases (a) Consecutive or obviously representative series of cases*  3) Selection of controls (a) Community control*  4) Definition of controls (a) No history of disease (endpoint)* | 1.) Comparability of cases and controls on the basis of the design or analysis:  (a) Study controls for serum 25(OH)D concentration CD.* | 1) Ascertainment of exposure: (a) Secure record *  2) Same method of ascertainment for cases and controls * (b) Yes  3) Non-Response rate (a) Same rate for both groups* | 8 | very low risk |
| Piatek-Guziewicz et al. 2017 | 29209126 | 1) Is the case definition adequate? (a) Yes, with independent validation *  2) Reperesentativeness of the cases (a) Consecutive or obviously representative series of cases*  3) Selection of controls (a) Community control*  4) Definition of controls (a) No history of disease (endpoint)* | 1.) Comparability of cases and controls on the basis of the design or analysis:  (a) Study controls for serum 25(OH)D concentration CD.* | 1) Ascertainment of exposure: (a) Secure record *  2) Same method of ascertainment for cases and controls * (b) Yes  3) Non-Response rate (a) Same rate for both groups* | 8 | very low risk |
| Tokgöz et al. 2018 | 29631542 | 1) Is the case definition adequate? (a) Yes, with independent validation *  2) Reperesentativeness of the cases (a) Consecutive or obviously representative series of cases*  3) Selection of controls (a) Community control*  4) Definition of controls (a) No history of disease (endpoint)* | 1.) Comparability of cases and controls on the basis of the design or analysis:  (a) Study controls for serum 25(OH)D concentration CD.* | 1) Ascertainment of exposure: (a) Secure record *  2) Same method of ascertainment for cases and controls * (b) Yes  3) Non-Response rate (a) Same rate for both groups* | 8 | very low risk |
| Işıkay et al. 2018 | 30102482 | 1) Is the case definition adequate? (a) Yes, with independent validation *  2) Reperesentativeness of the cases (a) Consecutive or obviously representative series of cases*  3) Selection of controls (a) Community control*  4) Definition of controls (a) No history of disease (endpoint)* | 1.) Comparability of cases and controls on the basis of the design or analysis:  (a) Study controls for serum 25(OH)D concentration CD.* | 1) Ascertainment of exposure: (a) Secure record *  2) Same method of ascertainment for cases and controls * (b) Yes  3) Non-Response rate (a) Same rate for both groups* | 8 | very low risk |
| Lionetti. et al. 2021 | 32889012 | 1) Is the case definition adequate? (a) Yes, with independent validation *  2) Reperesentativeness of the cases (a) Consecutive or obviously representative series of cases*  3) Selection of controls (a) Community control*  4) Definition of controls (a) No history of disease (endpoint)* | 1.) Comparability of cases and controls on the basis of the design or analysis:  (a) Study controls for serum 25(OH)D concentration CD.* | 1) Ascertainment of exposure: (a) Secure record *  2) Same method of ascertainment for cases and controls * (b) Yes  3) Non-Response rate (a) Same rate for both groups* | 8 | very low risk |
| Uyanıkoglu et al. 2021 |  | 1) Is the case definition adequate? (a) Yes, with independent validation *  2) Reperesentativeness of the cases (a) Consecutive or obviously representative series of cases*  3) Selection of controls (a) Community control*  4) Definition of controls (a) No history of disease (endpoint)* | 1.) Comparability of cases and controls on the basis of the design or analysis:  (a) Study controls for serum 25(OH)D concentration in CD.* | 1) Ascertainment of exposure: (a) Secure record *  2) Same method of ascertainment for cases and controls * (b) Yes  3) Non-Response rate (a) Same rate for both groups* | 8 | very low risk |
| Pedro et al.  2005 | 16278149 | 1) Is the case definition adequate? (a) Yes, with independent validation *  2) Reperesentativeness of the cases (a) Consecutive or obviously representative series of cases*  3) Selection of controls (a) Community control*  4) Definition of controls (a) No history of disease (endpoint)* | 1.) Comparability of cases and controls on the basis of the design or analysis:  (a) Study controls for VDR genotype in CD.* | 1) Ascertainment of exposure: (a) Secure record *  2) Same method of ascertainment for cases and controls * (b) Yes  3) Non-Response rate (a) Same rate for both groups* | 8 | very low risk |
| Rudko et al. 2008 |  | 1) Is the case definition adequate? (a) Yes, with independent validation *  2) Reperesentativeness of the cases (a) Consecutive or obviously representative series of cases*  3) Selection of controls (a) Community control*  4) Definition of controls (a) No history of disease (endpoint)* | 1.) Comparability of cases and controls on the basis of the design or analysis:  (a) Study controls for VDR genotype in CD.* | 1) Ascertainment of exposure: (a) Secure record *  2) Same method of ascertainment for cases and controls * (b) Yes  3) Non-Response rate (a) Same rate for both groups* | 8 | very low risk |
| Mårild et al. 2017 | 28686601 | 1) Is the case definition adequate? (a) Yes, with independent validation *  2) Reperesentativeness of the cases (a) Consecutive or obviously representative series of cases*  3) Selection of controls (a) Community control*  4) Definition of controls (a) No history of disease (endpoint)* | 1.) Comparability of cases and controls on the basis of the design or analysis:  (a) Study controls for VDR genotype in CD.* | 1) Ascertainment of exposure: (a) Secure record *  2) Same method of ascertainment for cases and controls * (b) Yes  3) Non-Response rate (a) Same rate for both groups* | 8 | very low risk |
| Vogelsang et al. 2000 | 10720115 | 1) Is the case definition adequate? (a) Yes, with independent validation *  2) Reperesentativeness of the cases (a) Consecutive or obviously representative series of cases*  3) Selection of controls (a) Community control*  4) Definition of controls (a) No history of disease (endpoint)* | 1.) Comparability of cases and controls on the basis of the design or analysis:  (a) Study controls for VDR genotype in CD.* | 1) Ascertainment of exposure: (a) Secure record *  2) Same method of ascertainment for cases and controls * (b) Yes  3) Non-Response rate (a) Same rate for both groups* | 8 | very low risk |
