# Supplementary material for: A meta-analysis suggests the association of reduced serum level of vitamin D and T-allele of Fok1 (rs2228570) polymorphism in the vitamin D receptor gene with celiac disease
Source: Front Nutr. 2023 Jan 19;9:996450. doi: 10.3389/fnut.2022.996450 (PMC9893277; doi:10.3389/fnut.2022.996450)
Supplement: Supplementary Table 3 — Mean difference of vitamin D [25(OH)D] concentration in serum. [file Table_3.docx]

**Supplementary Tables**

**Supplementary Table 3:** Mean difference of vitamin D [25(OH)D] concentration in serum of healthy controls and CD patients

| **Author, Year** | **Control** | | | **Cases** | | | **Mean difference** |
| --- | --- | --- | --- | --- | --- | --- | --- |
|  | **Mean** | **SD** | **Total** | **Mean** | **SD** | **Total** |  |
| Setty-Shah et al. 2014 | 26.14 | 26.03 | 49 | 29.81 | 27.22 | 18 | -3.67 |
| Nwosu et al. 2015 | 26.2 | 26.1 | 49 | 28.24 | 25.7 | 25 | -2.04 |
| Björck et al. 2017 | 30.88 | 3.3 | 142 | 26.32 | 4.8 | 71 | 4.56 |
| Piatek-Guziewicz et al. 2017 | 29.7 | 5.1 | 25 | 19.4 | 9 | 29 | 10.30 |
| Tokgöz et al. 2018 | 27.6 | 10.4 | 50 | 19.8 | 7.9 | 52 | 7.80 |
| Işıkay et al. 2018 | 14.33 | 3.8 | 268 | 12.39 | 12.8 | 226 | 1.94 |
| Lionetti. et al. 2021 | 31.6 | 13.7 | 131 | 25.3 | 8 | 131 | 6.30 |
| Uyanıkoglu et al. 2021 | 31.31 | 22.9 | 40 | 21.95 | 17.09 | 40 | 9.36 |
