# Supplementary material for: A meta-analysis suggests the association of reduced serum level of vitamin D and T-allele of Fok1 (rs2228570) polymorphism in the vitamin D receptor gene with celiac disease
Source: Front Nutr. 2023 Jan 19;9:996450. doi: 10.3389/fnut.2022.996450 (PMC9893277; doi:10.3389/fnut.2022.996450)
Supplement: Supplementary Table 4 — Allele frequencies of four VDR SNPs those were included in the study of healthy controls and CD patients. [file Table_4.docx]

**Supplementary Tables**

**Supplementary Table 4:** Allele frequencies of four VDR SNPs those were included in the study.

| **Gene name** | **Marker** | **Risk allele** | **Cases** | | **Control** | | **Author,Year** | **Ethnicity** |
| --- | --- | --- | --- | --- | --- | --- | --- | --- |
|  |  |  | **Allele** | **Total** | **Allele** | **Total** |  |  |
| VDR | Bsm1 rs1544410 | G | 52 | 78 | 98 | 176 | Pedro et al.  2005 | Spanish |
|  |  | G | 30 | 98 | 62 | 196 | Rudko et al. 2008 | Russian |
|  |  | G | 447 | 788 | 640 | 1096 | Mårild et al. 2017 | Norwegian |
|  | Apa1rs7975232 | C | 72 | 184 | 94 | 222 | Vogelsang et al. 2000 | Viennese |
|  |  | C | 45 | 78 | 77 | 176 | Pedro et al.  2005 | Spanish |
|  | Fok1rs2228570 | T | 32 | 78 | 55 | 176 | Pedro et al.  2005 | Spanish |
|  |  | T | 48 | 98 | 88 | 226 | Rudko et al. 2008 | Russian |
|  | Taq1 rs731236 | T | 77 | 184 | 94 | 222 | Vogelsang et al. 2000 | Viennese |
|  |  | T | 23 | 78 | 77 | 182 | Pedro et al.  2005 | Spanish |
